# Clinical characteristics and risk factors associated with bone erosion in patients with tophi

**DOI:** 10.1186/s42358-023-00336-w

**Published:** 2024-03-04

**Authors:** Zhuyi Ji, Yukai Huang, Ling Liang, Paifeng Lin, Xin Guo, Qidang Huang, Zhengping Huang, Shuyang Chen, Zhixiang Huang, Biao Wang, Lixin Huang, Shanmiao Sun, Weiming Deng, Tianwang Li

**Affiliations:** 1 The Affiliated Guangdong Second Provincial General Hospital of Jinan University, Guangzhou, 510632 China; 2 Department of Rheumatology and Immunology, Guangdong Second Provincial General Hospital, Guangzhou, 510317 China; 3 The Second Affiliated Hospital of Guangzhou Medical University, Guangzhou, 510260 China; 4 Department of Cardiology, Liuzhou People’ s Hospital, Affiliated of Guangxi Medical University, Liuzhou, 545006 China

**Keywords:** Gout, Tophi, Bone erosion, Characteristic, Risk factors

## Abstract

**Introduction:**

If a large amount of urate crystals is deposited in a joint cavity for an extended period of time, bone erosion will occur and gradually cause skeletal muscle necrosis and joint deformity. The aim of this study was to describe the clinical characteristics and factors associated with bone erosion in gout patients with tophi.

**Methods:**

A total of 210 gout patients with tophi were enrolled and divided into a bone erosion group (n = 135) and a non-bone erosion group (n = 75). Digital radiography (DR) was performed to detect bone erosion in the elbow, wrist, knee, ankle joints, interphalangeal and metatarsophalangeal joints. The clinical characteristics were recorded and compared between the two groups. Multivariate logistic regression analysis was conducted to explore the factors associated with bone erosion.

**Results:**

Compared with the non-bone erosion group, the bone erosion group had an older age, longer disease duration of gout and tophi, higher level of serum creatinine (sCr), higher proportion of drinking history and ulceration, and a lower glomerular filtration rate (GFR). Univariate logistic regression analysis results showed that sex, age, body mass index (BMI), gout duration, tophi duration, GFR, white blood cell (WBC) count, sCr level, smoking history, drinking history, and presence of ulceration were associated with bone destruction. Multivariable logistic regression analysis results indicated that tophi duration, drinking history, ulceration and sCr were positively and independently related to bone erosion.

**Conclusions:**

Tophi patients with bone erosion presented different clinical characteristics. Tophi duration, drinking history, ulceration and sCr were associated with bone erosion in gout patients with tophi.

**Supplementary Information:**

The online version contains supplementary material available at 10.1186/s42358-023-00336-w.

## Introduction

Gout is a prevalent chronic inflammatory arthritis disease characterized by the deposition of monosodium urate (MSU) crystals in joints, cartilage, synovial bursa, tendons or soft tissues [1]. It has been reported that the prevalence of gout ranges from 1 to 4% worldwide and that the incidence ranges from 0.1 to 0.3% [2]. The incidence and prevalence of gout have increased in recent years due to changes in lifestyle and diet [2, 3]. If left untreated, the frequency of acute attacks of gout increases, and deposition of urate crystals can lead to tophi and bone erosion, followed by joint damage, deformity and disability [4]. Tophi is a chronic inflammatory tissue surrounding the accumulation of MSU crystals [5]. Bone erosion is a widespread complication of tophaceous gout [6]. Bone destruction in gout is closely associated with MSU crystals and the soft tissue component of tophi [7]. It is necessary to explore other risk factors related to bone destruction in gout.

Some epidemic investigations showed that some higher risk factors for gout were found, including age, sex, alcohol consumption, diet, metabolic syndrome, hypertension, diabetes, and renal insufficiency [8, 9]. The incidence of subcutaneous tophi was closely associated with the duration of disease, a higher risk of cardiovascular events, renal insufficiency, kidney stones, hypertension, and higher mortality [10–12]. Mian Wu et al. detected bone destruction in gout patients by using ultrasound scans and explored the affecting factors related to bone erosion, and the results showed that age, duration of gout, the number of tophi, and synovial hypertrophy were the main connected factors [13]. Because of the close relationship between tophi and bone erosion, it is worthwhile to further explore the factors associated with bone destruction based on the populations of gout patients with tophi.

In our study, the participants in the two groups, bone erosion and non-bone erosion, were all gout patients with tophi. The clinical characteristics were recorded and compared between the two groups. To investigate the factors associated with bone erosion, we used multivariate logistic regression analysis.

## Materials and methods

### Patients

A total of 210 gout patients with tophi were recruited for the study from our hospital from April 2018 to July 2021. The presence of tophi was diagnosed by physical examination. Gout was classified based on the criteria of the 2015 American College of Rheumatology/European League Against Rheumatism (ACR/EULAR) [14]. Gout patients with other rheumatic diseases were excluded from our study, including those with rheumatoid arthritis, spondyloarthropathy, systemic lupus erythematosus, and osteoarthritis. The elbow, wrist, knee, ankle joints, interphalangeal and metatarsophalangeal joints were routinely scanned by digital radiography (DR) examination. Bone erosions were assessed by two experienced radiologists. Gout patients with tophi were further divided into a bone erosion group (n = 135) and a non-bone erosion group (n = 75). The study was approved by the EC office of Guangdong Second Provincial General Hospital (2021-KZ-131-01), and the whole process was conducted in accordance with the Declaration of Helsinki.

### Collection of clinical characteristics and laboratory data

Clinical characteristics and laboratory data, including sex, age, weight, height, smoking history, drinking history, gout duration, tophi duration, ulceration, complications, glomerular filtration rate (GFR), white blood cell (WBC) count, platelet (PLT) count, hemoglobin (HGB), alanine aminotransferase (ALT), serum uric acid (sUA), serum creatinine (sCr), C-reactive protein (CRP) and erythrocyte sedimentation rate (ESR) were collected and measured. Smoking history was defined as current smoking or past smoking with less than three years of smoking cessation. Drinking history was defined as consumption of liquor > 40 g/d. The assessment of ulceration was conducted according to Wagner’s classification and grade one or higher was defined as ulceration. The GFR was measured by emission computed tomography (ECT).

### Statistical analysis

SPSS 23.0 software was used for database management and statistical analysis. Continuous variables are described as the mean ± standard deviation (mean ± SD), and categorical variables are described as frequencies and percentages. For continuous variables, independent-samples t tests were used to compare the differences between the groups. The chi-square test and Fisher’s exact test were used for categorical variables. Univariate binary logistic regression was performed to identify statistically significant correlations between bone erosion and each potential risk factor (P<0.1). Variables with significant differences were defined as candidate factors for multivariate logistic regression analysis. To evaluate the independent risk factors of for bone erosion, a further multivariable logistic regression model using the forward stepwise likelihood ratio method was fitted with candidate factors, with an entry probability of 0.05 and removal of 0.10. Odds ratios (ORs) and 95% confidence intervals were calculated for the multivariate logistic model.

## Results

### Comparison of clinical characteristics between the two groups

Compared with the non-bone erosion group, the bone erosion group had an older age, longer disease duration of gout and tophi, higher level of sCr, higher proportion of drinking history and ulceration, and a lower GFR. There were no significant differences between the two groups regarding sex, body mass index (BMI), hypertension, diabetes, smoking history, WBC count, PLT count, HGB, ALT, sUA, CRP, or ESR **(**Table 1**)**.

**Table 1:** Comparison of clinical characteristics between the two groups

	Bone erosion	Non-Bone erosion	*P* value
**N (male)**	135(132)	75(69)	0.104
**Age (year)**	52.40 ± 14.32	46.33 ± 13.57	**0.001**
**BMI (kg/m2)**	24.66 ± 3.49	25.60 ± 4.21	0.084
**Gout duration (year)**	11.80 ± 6.31	9.13 ± 5.47	**0.001**
**Tophi duration (year)**	5.44 ± 3.86	3.55 ± 4.12	**<0.001**
**GFR (ml/min/1.73m2)**	61.61 ± 24.10	68.85 ± 21.98	**0.033**
**Hypertension, n (%)**	39	39	0.983
**Diabetes, n (%)**	9	15	0.199
**Smoking history, n (%)**	52	39	0.067
**Drinking history, n (%)**	41	23	**0.008**
**Ulceration, n (%)**	29	12	**0.005**
**WBC count (10^9/mL)**	10.79 ± 12.16	8.81 ± 2.72	0.261
**PLT count (10^9/mL)**	312.67 ± 112.32	297.48 ± 95.17	0.659
**HGB (g/L)**	128.04 ± 24.14	130.59 ± 19.23	0.708
**ALT (U/L)**	31.13 ± 25.07	32.84 ± 24.22	0.367
**sUA (mg/dl)**	9.53 ± 1.94	9.45 ± 2.34	0.756
**sCr (mg/dl)**	1.54 ± 0.55	1.33 ± 0.39	**0.001**
**CRP (mg/L)**	30.47 ± 38.03	32.26 ± 41.79	0.919
**ESR (mm/h)**	48.08 ± 36.50	47.86 ± 32.69	0.664

Abbreviations: BMI, body mass index; GFR, glomerular filtration rate; WBC, white blood cell; PLT, platelet; HGB, hemoglobin; ALT, alanine aminotransferase; sUA, serum uric acid; sCr, serum creatinine; CRP, C-reactive protein; ESR, erythrocyte sedimentation rate

### Univariate logistic regression analysis of factors associated with bone erosion

Univariate logistic regression analysis results showed that sex, age, BMI, gout duration, tophi duration, GFR, WBC count, sCr, smoking history, drinking history, and presence of ulceration were associated with bone erosion **(**Table 2**)**.

**Table 2:** Univariate logistic regression analysis of factors associated with bone erosion

	β	OR (95% CI)	*P* value
**N (male)**	-1.342	0.261(0.063–1.077)	**0.063**
**Age**	0.031	1.031(1.010–1.053)	**0.004**
**BMI**	-0.067	0.936(0.867–1.009)	**0.085**
**Gout duration**	0.081	1.085(1.028–1.145)	**0.003**
**Tophi duration**	0.156	1.169(1.062–1.288)	**0.002**
**GFR**	-0.013	0.987(0.975–0.999)	**0.035**
**Hypertension**	-0.006	0.994(0.557–1.774)	0.983
**Diabetes**	-0.566	0.568(0.237–1.358)	0.203
**Smoking history**	0.535	1.708(0.962–3.034)	**0.068**
**Drinking history**	0.853	2.346(1.236–4.450)	**0.009**
**Ulceration**	1.092	2.979(1.353–6.562)	**0.007**
**WBC count**	0.080	1.084(0.994–1.182)	**0.069**
**PLT count**	0.001	1.001(0.999–1.004)	0.323
**HGB**	-0.005	0.995(0.982–1.008)	0.431
**ALT**	-0.003	0.997(0.986–1.009)	0.631
**sUA**	<0.001	1.000(0.998–1.003)	0.794
**sCr**	0.013	1.013(1.004–1.022)	**0.005**
**CRP**	-0.001	0.999(0.992–1.006)	0.752
**ESR**	<0.001	1.000(0.992–1.008)	0.965

Note: Analysis was performed using multivariate binary logistic regression

Abbreviations: CI, confidence interval; BMI, body mass index; GFR, glomerular filtration rate; WBC, white blood cell; PLT, platelet; HGB, hemoglobin; ALT, alanine aminotransferase; sUA, serum uric acid; sCr, serum creatinine; CRP, C-reactive protein; ESR, erythrocyte sedimentation rate

### Multivariable logistic regression analysis of factors associated with bone erosion

Multivariable logistic regression analysis results indicated that tophi duration (OR = 1.112, 95% CI = 1.012–1.222, *P* = 0.027), drinking history (OR = 2.263, 95% CI = 1.135–4.514, *P* = 0.020), ulceration (OR = 2.466, 95% CI = 1.051–5.787, *P* = 0.038), and sCr (OR = 1.015, 95% CI = 1.005–1.025, *P* = 0.003) were independently and positively associated with bone erosion **(**Table 3**)**.

**Table 3:** Multivariable logistic regression analysis of factors associated with bone erosion

	β	OR (95% CI)	*P* value
**Tophi duration**	0.106	1.112(1.012–1.222)	**0.027**
**Drinking history**	0.817	2.263(1.135–4.514)	**0.020**
**Ulceration**	0.903	2.466(1.051–5.787)	**0.038**
**sCr**	0.015	1.015(1.005–1.025)	**0.003**

Note: Analysis was performed using univariate binary logistic regression

Abbreviations: CI, confidence interval; sCr, serum creatinine

## Discussion

There have been many previous studies on the formation mechanism, clinically relevant risk factors, and ulceration associated with tophi [15–19]. In recent years, bone destruction due to gout has gradually attracted much attention [20–23] and its relevant clinical features and risk factors warrant further investigation. Our study showed that tophi patients with bone erosion presented different clinical characteristics. Tophi duration, drinking history, ulceration and sCr were associated with bone erosion in tophi patients.

With the increased rates of uncontrolled sUA, the incidence and prevalence of bone erosion have increased in recent years. It is necessary to explore other risk factors related to bone destruction in gout. Mian Wu et al. detected bone destruction in gout patients by using ultrasound scans and explored the factors connected with bone destruction, and the results showed that age, duration of gout, number of tophi, and synovial hypertrophy were the main correlative factors [13]. However, ultrasound has poor penetration into bone and is vulnerable to interference from other tissues, leading to inaccurate diagnoses. There are some studies demonstrating that ultrasound did not show satisfactory diagnostic accuracy in the diagnosis of bone erosion in rheumatoid arthritis (RA) [24]. The values of sensitivity (from 0.19 to 1.00) and specificity (from 0.44 to 1.00) of ultrasound in the bone erosion diagnosis had been observed to be far from consistent in different studies [25, 26]. In addition, ultrasound diagnosis also depends more on the technology and ability of the tester. DR for the assessment of bone erosions is characterized by high resolution, particularly for bone structures. Our study evaluated both upper-limb and lower-limb joints and further explored the clinical features of ulceration. In addition, the GFR was measured by ECT, which is clearer and more intuitive. More importantly, our subjects were tophi patients, while those of the Mian Wu et al. study were gout patients, therefore, we could find reliable risk factors more easily. In the present study, we further explored the factors associated with bone erosion based on populations of gout patients with tophi. The results showed that tophi duration, drinking history, ulceration and sCr were associated with bone erosion in gout patients with tophi. This is different from the previous study [13], which can deepen our understanding of the risk factors for bone destruction.

The age was older and the duration of gout was longer in patients with bone erosion, but those variables were not shown to be risk factors for bone erosion. Sex, BMI, sUA, CRP, and ESR were reported to have a close relationship with gout [27], but they were not significantly different between the bone erosion group and non-bone erosion groups. We knew that obesity and sUA were important risk factors for gout, however, we did not find that BMI and urate deposition on the cartilage surface resulted in an increased incidence of erosion, which was consistent with the previous study. It is possible that socioeconomic factors can affect BMI, including family genetics, eating habits, exercise habits, and occupation category. In addition, due to the relationship between serum uric acid levels and bone destruction, some urate crystals could be asymptomatic in the absence of active inflammatory symptoms, possibly due to the formation of neutrophil extracellular traps [28]. McQueen et al. found that erosions were not connected with bone edema or synovitis but were strongly associated with tophi in a prospective magnetic resonance imaging (MRI) study [29]. With the formation of tophi, foreign body granulomas composed of mononuclear and multinucleated macrophages surrounding the deposition of MSU are present. Increased osteoclast-mediated bone resorption and impaired osteoblast-mediated bone formation indicate very strong evidence of disturbed bone remodeling in tophaceous arthritis [30]. Lee SJ et al. demonstrated that receptor activator nuclear factor-kappa B ligand (RANKL)-expressing T cells and TRAP + osteoclasts were present within gouty tophus tissues [31]. Bone erosion affects the activities of daily living of patients with gout and even aggravates the socioeconomic burden. Taken together, these results all suggested that early prevention, diagnosis, and treatment of tophi is important to prevent the formation of bone erosions. Prolonged tophi duration was correlated with the number and volume of tophi, which could aggravate bone destruction. It also suggested the importance of early detection of tophi by computed tomography, ultrasonography, and MRI. In addition, ulceration of tophi was the most forceful factor associated with bone erosion, with an OR of 2.466. This may be related to the large number of inflammatory factors caused by the ulcers. Granulomatous responses in bones and joints lead to erosions and bone destruction, with proresorptive cytokines such as IL-1β, IL-6, and TNF-α driving the formation of tophi to erode the underlying bone. Under normal physiological conditions, there is a balance between resorbed old bone and formed new bone. However, this balance between osteoclasts and osteoblasts is disrupted by bone destruction with gouty arthritis. Macrophage colony stimulating factor (M-CSF) and RANKL-activated receptors play a key role in the environment of erosive bone destruction [31, 32]. The relationship of drinking history and sCr with bone erosion may be because alcoholism and renal impairment lead to the uncontrolled sUA, hindering the reduction of tophi.

In addition, some limitations should be accounted for in our study. First, tophi were not pathologically confirmed due to the invasiveness of the required investigations. Second, the number of patients was relatively small, and this was a single-center study. Therefore, it is necessary to increase the sample size and conduct multicenter research to confirm these results.

In conclusion, we found that there were some different clinical characteristics between bone erosion and non-bone erosion in gout patients with tophi. Tophi duration, drinking history, ulceration and sCr were independently associated with erosions in gout patients with tophi. Prolonged duration with tophi, chronic alcohol consumption, presence of tophi ulceration and elevated creatinine increase the risk of bone erosion over tophi. This sheds further light on the links between crystal deposition, a daily diet and joint damage in tophi patients. Therefore, we should pay specially attention to patients with risk factors for bone erosion over tophi.

## Electronic supplementary material

Below is the link to the electronic supplementary material.

Supplementary Material 1

Supplementary Material 2

Supplementary Material 3

## Data Availability

All data used during the study are available from the corresponding author by request.
