# Supplementary material for: Clinical characteristics and risk factors associated with bone erosion in patients with tophi
Source: Adv Rheumatol. 2024 Mar 4;64:18. doi: 10.1186/s42358-023-00336-w (PMC13488662; doi:10.1186/s42358-023-00336-w)
Supplement: Supplementary file 1 [file 42358_2023_336_MOESM3_ESM.pdf]

Changes to authorship

Manuscript No: ADRH-D-23-00020R1

Article entitled: Clinical characteristics and factors associated with bone erosion in gout patients with tophi

Author: Zhuyi Ji[#1,2], Yukai Huang[#2], Ling Liang[#3], Paifeng Lin[#1,2], Xin Guo[#2], Qidang Huang[2], Zhengping Huang[2], Shuyang Chen[2], Zhixiang Huang[2], Biao Wang[4], Lixin Huang[2], Shanmiao Sun[2], Weiming Deng[*2], Tianwang Li[*1,2]

1. The Affiliated Guangdong Second Provincial General Hospital of Jinan University, Guangzhou 510632, China

2. Department of Rheumatology and Immunology, Guangdong Second Provincial General Hospital, Guangzhou 510317, China

3. The Second Affiliated Hospital of Guangzhou Medical University, Guangzhou 510260, China

4. Department of Cardiology, Liuzhou People' s Hospital, Affiliated of Guangxi Medical University, Liuzhou 545006, China

#Zhuyi Ji, Yukai Huang, Ling Liang, Paifeng Lin, and Xin Guo contributed equally to this work

*Corresponding author: Weiming Deng (15088097855@163.com) and Tianwang Li (litian-wang@163.com).

We would like to change the author order and add three authors due to the great contribution of Paifeng Lin, Zhixiang Huang and Biao Wang to this article during the revision process. Zhixiang Huang and Biao Wang are two recipients of our article fund sources. We have checked the list of fund sources and found that there were three previously overlooked fund sources. We have updated fund sources. We were really sorry for our careless mistakes.

After consultations, all the authors agreed with the addition of the author and the rearrangement of the names. All the authors signed in changes to authorship form.

Below are the signatures of all authors.

Signed: Zhuyi Ji, Yukai Huang, Ling Liang, Paifeng Lin, Xin Guo, Qidang Huang, Zhengping Huang, Shuyang Chen, Zhixiang Huang, Biao Wang, Lixin Huang, Shanmiao Sun, Weiming Deng, Tianwang Li

Date: 14 October, 2023
