# Supplementary material for: Clinical characteristics and risk factors associated with bone erosion in patients with tophi
Source: Adv Rheumatol. 2024 Mar 4;64:18. doi: 10.1186/s42358-023-00336-w (PMC13488662; doi:10.1186/s42358-023-00336-w)
Supplement: Supplementary file 2 [file 42358_2023_336_MOESM1_ESM.docx]

Dear reviewers,

On behalf of all the contributing authors, I would like to express our sincere appreciations of reviewers’ constructive comments concerning our article entitled “**Clinical characteristics and factors associated with bone erosion in gout patients with tophi**” (Manuscript No: ADRH-D-23-00020R1). These comments are very valuable and help to improve our article. Based on the reviewers' comments, we have revised and modified our manuscript. In the revised version, changes to our manuscript are highlighted in the file using blue text. Point-by-point responses to the reviewer are listed below this letter.

Reviewer reports

Reviewer #1

Is the statement that "ultrasound has poor penetration into bone and is vulnerable to other tissues" supported by the literature? Ultrasonography does not penetrate the bone, but it can detect erosions with great sensitivity, identifying the loss of continuity on the surface of the bone cortex.

Response: Thanks for your valuable comments. We add this point in the Discussion section **as shown in [lines] and below.** There are some studies demonstrating that ultrasound did not show satisfactory diagnostic accuracy in the diagnosis of bone erosion in rheumatoid arthritis (RA)^[1]^. The values of sensitivity (from 0.19 to 1.00) and specificity (from 0.44 to 1.00) of ultrasound in the bone erosion diagnosis had been observed to be far from consistent in different studies^[2, 3]^. In addition, ultrasound diagnosis also depends on the technology and ability of the tester.

About the conclusion: "These results indicated that reasonable diet, early diagnosis and treatment of tophi and ulceration may be essential to prevent bone erosion." Although the statement is logical, it cannot be made based on the results of this study, which is descriptive and not longitudinal.

Response: Thanks for your valuable comments. We modified this section **as shown in [lines] and below.** Prolonged duration with tophi, chronic alcohol consumption, presence of tophi ulceration and elevated creatinine increase the risk of bone erosion over tophi. This sheds further light on the links between crystal deposition, a daily diet and joint damage in tophi patients. Therefore, we should pay specially attention to patients with risk factors for bone erosion over tophi.

References

[1]	Tang H, Qu X, Yue B. Diagnostic test accuracy of magnetic resonance imaging and ultrasound for detecting bone erosion in patients with rheumatoid arthritis [J]. Clin Rheumatol, 2020, 39(4): 1283-93.

[2]	Peluso G, Bosello S L, Gremese E, et al. Detection of bone erosions in early rheumatoid arthritis: 3D ultrasonography versus computed tomography [J]. Clin Rheumatol, 2015, 34(7): 1181-6.

[3]	Kawashiri S-y, Suzuki T, Okada A, et al. Musculoskeletal ultrasonography assists the diagnostic performance of the 2010 classification criteria for rheumatoid arthritis [J]. Modern Rheumatology, 2013, 23(1): 36-43.
