# Supplementary material for: Clinical characteristics and risk factors associated with bone erosion in patients with tophi
Source: Adv Rheumatol. 2024 Mar 4;64:18. doi: 10.1186/s42358-023-00336-w (PMC13488662; doi:10.1186/s42358-023-00336-w)
Supplement: Supplementary file 3 [file 42358_2023_336_MOESM2_ESM.docx]

Dear Editor:

Thanks for providing us with this great opportunity to submit a revised version of our article entitled “**Clinical characteristics and factors associated with bone erosion in gout patients with tophi**” (Manuscript No: ADRH-D-23-00020R1). We appreciate the detailed and constructive comments provided by the reviewers. Based on the reviewers' comments, we have revised and modified our manuscript. In the revised version, changes to our manuscript are highlighted in the file using blue text. Point-by-point responses to the reviewer are listed in the rebuttal letter.

We would like to change the author order and add three authors due to the great contribution of Paifeng Lin, Zhixiang Huang and Biao Wang to this article during the revision process. Zhixiang Huang and Biao Wang are two recipients of one of our article fund sources. We have checked the list of fund sources and found that there were three previously overlooked fund sources. We have updated fund sources. We were really sorry for our careless mistakes. The revised author list would be as follows: Zhuyi Ji, Yukai Huang, Ling Liang, Paifeng Lin, Xin Guo, Qidang Huang, Zhengping Huang, Shuyang Chen, Zhixiang Huang, Biao Wang, Lixin Huang, Shanmiao Sun, Weiming Deng, Tianwang Li.

After consultations, all the authors agreed with the addition of the author and the rearrangement of the names. All the authors signed in changes to authorship form.

We apologize for any inconvenience this change may cause and kindly request your approval for this authorship modification.

Thank you for your consideration. I look forward to hearing from you.

Sincerely,

Tianwang Li
